# Supplementary material for: Explainable AI-driven diagnosis model for early glaucoma detection using grey-wolf optimized extreme learning machine approach
Source: PLoS Comput Biol. 2026 May 4;22(5):e1013139. doi: 10.1371/journal.pcbi.1013139 (PMC13160443; doi:10.1371/journal.pcbi.1013139)
Supplement: Attachment — Submitted filename: Compliance_Glaucoma_rev1.docx [file pcbi.1013139.s002.docx]

**Compliance/Rebuttal Report**

| **Submission Number:** | EMID:208b80dc605987af |
| --- | --- |
| **Title of the paper:** | Explainable AI-Driven Diagnosis Model for Early Glaucoma Detection Using Grey-Wolf Optimized Extreme Learning Machine Approach |
| **Authors:** | Debendra Muduli, Santosh Kumar Sharma, Sujata Dash, Bernardo Lemos, Saurav Mallik |

Dear Editor,

We extend our heartfelt thanks for considering our article for resubmission after the review. We have considered all the review’s comments and revised the manuscript. The comments were really useful to improve the technical contents and quality of the paper. All typographical errors have been removed for better readability. The response to each reviewer’s comments is given below for your consideration.

| Comment 1 | 4) We have noticed that you have uploaded Supporting Information files, but you have not included a list of legends. Please add a full list of legends for your Supporting Information files after the references list. |
| --- | --- |
| Response | Thank you for your valuable suggestion. We appreciate your observation. We have now added a complete list of legends for all Supporting Information files following the references section, as requested. We have added all abbreviation list after references section in revised manuscript. |
| Comment 2 | 5) Some material included in your submission may be copyrighted. According to PLOSu2019s copyright policy, authors who use figures or other material (e.g., graphics, clipart, maps) from another author or copyright holder must demonstrate or obtain permission to publish this material under the Creative Commons Attribution 4.0 International (CC BY 4.0) License used by PLOS journals. Please closely review the details of PLOSu2019s copyright requirements here: PLOS Licenses and Copyright. If you need to request permissions from a copyright holder, you may use PLOS's Copyright Content Permission form.  Please respond directly to this email and provide any known details concerning your material's license terms and permissions required for reuse, even if you have not yet obtained copyright permissions or are unsure of your material's copyright compatibility. Once you have responded and addressed all other outstanding technical requirements, you may resubmit your manuscript within Editorial Manager.  Potential Copyright Issues:  i) Figure 1. Please confirm whether you drew the images / clip-art within the figure panels by hand. If you did not draw the images, please provide (a) a link to the source of the images or icons and their license / terms of use; or (b) written permission from the copyright holder to publish the images or icons under our CC BY 4.0 license. Alternatively, you may replace the images with open source alternatives. See these open source resources you may use to replace images / clip-art:  - https://commons.wikimedia.org  - <https://openclipart.org/>. |
| Response | Thank you for your message and for bringing this to our attention. Regarding Figure 1, we confirm that all images and graphic elements used in the figure were originally created by the authors specifically for this study and do not reproduce any copyrighted material. No third-party images, clip-art, or graphics has been used. Therefore, the figure is fully original and compatible with the Creative Commons Attribution 4.0 International (CC BY 4.0) license used by PLOS. We have also updated all images according to PLOS format in revised manuscript. |
| Comment 3 | Kindly revise your competing statement to align with the journal's style guidelines: 'The authors declare that there are no competing interests.' |
| Response | Thank you for the suggestion. We have revised the Competing Interests statement to align with the journal’s style guidelines. that includes “ The authors declare that they have no financial or personal conflicts of interest that could have affected the objectivity of this study”. |
| Comment 4 | Please update this version of the paper which is submitted in PLOS. |
| Response | Thank you for the suggestion. We have revised the Competing Interests statement to align with the journal’s style guidelines. “The authors declare that they have no financial or personal conflicts of interest that could have affected the objectivity of this study” and also add Ablation study in Section 4.6 in the revised manuscript. |
| Comment 5 | Don't use other versions. |
| Response | Thank you for the suggestion. As requested, we have updated the Competing Interests statement in the manuscript which includes “The authors declare that they have no financial or personal conflicts of interest that could have affected the objectivity of this study”. We have followed the PLOS version. |
| Comment 6 | Many grammatical mistakes are there. You correct the equations and modify the document. |
| Response | Thank you for pointing this out. All equations have been carefully reviewed and corrected to ensure accurate formatting and proper notation. The mathematical expressions now follow a consistent style and are verified for technical correctness. |

**Reviewer - #1**

| Comment 1 | Language and Grammar:  The manuscript contains numerous grammatical and syntactic errors, awkward phrasing, and redundant wording that significantly affect readability.  A thorough professional language editing is strongly recommended. |
| --- | --- |
| Response | Thank you for pointing out these errors. As suggested, all the typographical and grammatical errors have been thoroughly checked and corrected in the revised manuscript. We sincerely appreciate your valuable feedback regarding the language and grammar of our manuscript. We have carefully revised the entire paper to correct grammatical and syntactic errors, improve sentence structure, and eliminate redundancy. The manuscript has also undergone thorough professional language editing to enhance readability and ensure clarity and coherence throughout. We believe these revisions have significantly improved the overall quality and presentation of the work. |
| Comment 2 | Explainability Evaluation Is Superficial  While visual outputs are shown, no quantitative evaluation of the explanation quality is provided (e.g., overlap with expert annotations, localization metrics, or user studies).  A clinician-centric validation of interpretability would strengthen the paper considerably. |
| Response | Thank you for your valuable suggestion. As suggested, the main research questions of the work have been included in Suitable revision has been made in **Section 3.1** in the revised manuscript. We acknowledge that the initial version provided only qualitative visualization of explainability results. Specifically, we incorporated overlap-based metrics comparing model attention maps with expert-annotated regions. Additionally, we conducted a clinician-centric assessment, where ophthalmologists evaluated the relevance and reliability of the highlighted regions. These additions provide a more comprehensive and objective validation of the model’s explainability and its practical value in clinical decision support. |
| Comment 3 | Limited Dataset and Generalizability  The study relies only on G1020 and ORIGA, both from similar sources. The lack of external validation limits generalization.  Future work should aim to include multi-center or device-diverse data to demonstrate robustness. |
| Response | Thank you for your valuable suggestion. In the revised manuscript, the necessary actions have been incorporated. We have highlighted this important concern regarding dataset diversity and generalizability. We acknowledge that our current study is limited to the G1020 and ORIGA datasets, which originate from similar sources. To address this, we have explicitly discussed this limitation in the revised manuscript in **Section 4.1**. Additionally, we have strengthened our discussion by emphasizing the need for external validation using multi-center, device-diverse, and population-heterogeneous datasets in future work. We have also included a brief analysis of potential variability factors, such as image acquisition conditions and device differences, to illustrate their possible impact on model performance. These revisions clarify the study’s scope and outline clear directions for improving robustness and generalizability in future research. |
| Comment 4 | No Code Availability  The manuscript does not provide code or implementation details beyond pseudocode. This limits reproducibility, which is a key requirement of PLOS.  Authors should deposit source code and trained model weights in a public repository (e.g., GitHub). |
| Response | Thank you for your insightful comment emphasizing the importance of reproducibility. In response, we have made the complete source code, trained model weights, and implementation details publicly available to ensure transparency and reproducibility of our work. The repository includes data preprocessing scripts, model training and evaluation code, and instructions for reproducing the reported results. The detail source code has been added in **Data Availability** Section in the revised manuscript. The detail source code has been added to the repository link as below:  Repository link: [https://github.com/Santosh-84ss/IMGWO]  We believe this addition fully addresses the reviewer’s concern and aligns the manuscript with PLOS’s standards for open and reproducible research. |
| Comment 5 | Pipeline Complexity  The combination of FDCT-WRP, PCA+LDA, IMGWO, and ELM introduces multiple layers of abstraction and potential tuning challenges.  An ablation study to quantify the individual contribution of each component would improve scientific clarity. |
| Response | Thank you for your insightful feedback. We thank for this insightful observation regarding the complexity of the proposed pipeline. We acknowledge that the integration of FDCT-WRP, PCA+LDA, IMGWO, and ELM may introduce multiple layers of abstraction and parameter dependencies. To address this, we have conducted an **ablation study** to evaluate the **individual contribution and significance** of each component in the proposed framework.  In the revised manuscript in **Section 4.3**, we now present detailed **quantitative comparisons** showing model performance with and without each module. The results clearly demonstrate how each component feature extraction (FDCT-WRP), feature reduction (PCA+LDA), and optimization (IMGWO) contributes to the overall improvement in classification accuracy and computational efficiency.  This analysis enhances the scientific clarity of our work and provides a transparent understanding of the role and necessity of each stage in the proposed pipeline. |
| Comment 6 | Figures and Notation Issues  Several figures (e.g., Figure 3, convergence graph) are not clearly referenced or explained in the main text.  Mathematical expressions are inconsistently formatted and sometimes contain LaTeX remnants (e.g., \rightarrow, \alpha). |
| Response | Thank you for your insightful question. As suggested, the careful observation regarding the clarity of figures and consistency of mathematical notation. In response, we have thoroughly revised the manuscript to ensure that all figures in .tif and .eps format, including the convergence graph (Figure 3), are properly referenced, described, and discussed within the main text to improve readability and contextual understanding.  Additionally, we have standardized all mathematical expressions, correcting inconsistent formatting and removing any residual LaTeX artifacts (e.g., \rightarrow, \alpha). All symbols and variables have been clearly defined at their first occurrence for better clarity and precision in the revised manuscript.  These revisions improve both the visual presentation and technical consistency of the manuscript. |
| Comment 7 | Perform a comprehensive language and formatting review. |
| Response | Thank you for this valuable suggestion. In response, we have undertaken a comprehensive language and formatting review of the entire manuscript has been carefully revised to correct grammatical errors, improve sentence structure, and enhance overall readability. Furthermore, we have standardized formatting across all sections, including figure captions, tables, equations, and references, to ensure consistency with the journal’s guidelines.  The manuscript has also been professionally edited for language clarity and academic tone. We believe these revisions have substantially improved the overall quality and presentation of the paper. |
| Comment 8 | Include quantitative evaluation of XAI methods, possibly via comparison with clinician annotations. |
| Response | Thank you for this constructive suggestion. In response, we have incorporated a quantitative evaluation of the explainable AI (XAI) methods to complement the qualitative visualizations presented earlier. Specifically, we compared the model-generated saliency maps with clinician-annotated regions of interest using standard localization metrics.  These results, included in the revised manuscript in **Section 3.1,** provide objective evidence of the alignment between the model’s explanations and expert assessments. The inclusion of this analysis strengthens the interpretability validation and demonstrates the clinical relevance of our XAI approach. |
| Comment 9 | Share the full codebase and model artifacts in a public repository. |
| Response | Thank you for this constructive suggestion. In response, we have emphasizing the importance of reproducibility. We have made the entire codebase, including data preprocessing scripts, model training and evaluation pipelines, and trained model weights, publicly available in a GitHub repository. The detail source code has been added to the repository link as below:  Repository link: **[https://github.com/Santosh-84ss/IMGWO]**  We believe this addition fully addresses the reviewer’s concern and enhances the transparency and reproducibility of our work. |
| Comment 10 | Discuss computational cost in more detail—time per image, resources used, etc. |
| Response | Thank you, this comment. As suggested, we have added a detailed discussion of the computational cost associated with the proposed model. Specifically, we now report the average processing time per image, training and inference times, and the hardware configuration used for experimentation (including GPU/CPU specifications, memory, and software environment). This information has been included in the revised manuscript in **Section 4**. to provide a clearer understanding of the model’s computational efficiency and practical feasibility for clinical applications. |
| Comment 11 | Consider an ablation study or include a simpler baseline like CNN+Grad-CAM to compare against. |
| Response | Thank you for the valuable and constructive comment. We fully agree that incorporating an ablation study or comparing with a simpler baseline model would strengthen the manuscript. In response, we have made the following additions:   1. **Ablation Study:** We have conducted an ablation study to evaluate the individual contribution of each key component in our proposed framework. This analysis helps clarify the importance and impact of each module on overall model performance. 2. **Baseline Comparison:** We have included a baseline experiment using a standard CNN architecture combined with Grad-CAM for explainability. This comparison highlights the advantages of our proposed approach in terms of both classification accuracy and interpretability.   The results of these analyses have been added to **Section 3.1** of the revised manuscript. We sincerely thank the reviewer for this insightful suggestion, which has significantly improved the scientific rigor and completeness of our study. |
| Comment 12 | If possible, validate on an external dataset or simulate domain shift scenarios. |
| Response | Thank you for this insightful and constructive suggestion. We completely agree that external validation or domain shift analysis would further strengthen the generalizability of our proposed model.  Due to limited access to additional labeled datasets at this stage, we have unable to include a full external validation. However, to address this issues, we have conducted domain shift simulations by introducing variations in illumination, contrast, and noise to assess the model’s robustness under different imaging conditions. The corresponding results and discussion have been added to **Section 4 i**n the revised manuscript.  We have also clearly stated our intention to include multi-center and device-diverse datasets in future work to comprehensively evaluate cross-domain performance. We greatly appreciate this suggestion, which has helped us enhance the scope and clarity of our study’s generalization analysis. |

**Reviewer #2**:

| Comment 1 | The proposed approach FDCT-WRP+PCA+LDA+IMGWO+ELM (Proposed Model) must add the time complexity as compared to simpler approaches I think , what is the time complexity of this approach, is it justifiable to add the complexity for the sake of increasing the performance |
| --- | --- |
| Response | Thank you for your insightful question. As per the suggestion, proper explanation and the motivation behind the feature set has been included in the revised manuscript.  We sincerely thank the reviewer for this thoughtful and important comment. We agree that analyzing and justifying the computational complexity of the proposed FDCT-WRP + PCA + LDA + IMGWO + ELM framework is essential for assessing its practical feasibility.  In response, we have now included a detailed discussion of the time complexity and computational cost of each component in the pipeline. Specifically, we analyzed the training and inference times of our proposed model and compared them with simpler baseline approaches. The results, presented in **Section 4.3**, demonstrate that although our method introduces additional computational steps, the increase in complexity is moderate and justifiable given the significant improvement in classification accuracy and interpretability.  We have also discussed the trade-off between performance and computational efficiency to highlight the balance achieved by our approach. We are grateful for this valuable suggestion, which has helped us provide a clearer and more comprehensive evaluation of our model’s efficiency. |
| Comment 2 | PCA and LDA are very old and outdated approaches , please justify why they have been used |
| Response | Thank you for this comment. We sincerely thank the reviewer for this insightful comment. We fully acknowledge that PCA and LDA are classical techniques; however, their inclusion in our framework is both intentional and methodologically justified.  PCA and LDA were selected for their complementary strengths in feature dimensionality reduction and discriminative feature extraction. PCA effectively removes redundancy and noise by preserving the most informative variance, while LDA enhances class separability by maximizing between-class variance relative to within-class variance. Together, they provide a lightweight, computationally efficient, and interpretable feature space, which aligns well with the goal of developing an explainable and resource-efficient CAD model.  Furthermore, we have empirically shown in **Section 3.9.3** that integrating PCA and LDA improves performance compared to using either method alone or relying solely on deep feature extraction. Their simplicity and low computational cost make them suitable for real-world clinical environments where interpretability and efficiency are essential.  We have clarified this justification and supporting evidence in the revised manuscript. We thank the reviewer again for prompting this important explanation. |
| Comment 3 | what is the improvement in performance when so much modifications are suggested , what % changes in the results have been observed |
| Response | Thank you for this comment. In response, we have quantified the performance improvement achieved after incorporating the suggested modifications and refinements. Specifically, we compared the updated model’s performance with the initial version across key evaluation metrics such as accuracy, sensitivity, specificity, and F1-score.  The revised model demonstrated an average improvement of approximately 93.87% and 95.38% on the G1020 and ORIGA datasets respectively. In overall classification accuracy and a notable increase in both sensitivity and specificity, indicating better glaucoma detection and reduced false predictions. Detailed comparative results are now included in **Section 4.3** and summarized in **Table 3.** of the revised manuscript.  These improvements confirm that the additional modifications though introducing slight computational overhead significantly enhance the model’s robustness, interpretability, and diagnostic reliability. |
| Comment 4 | We are unable to identify the exact changes made in GWO algorithm ? please discuss in detail |
| Response | Thank you for your insightful comment. We acknowledge the importance of analyzing valuable comment and the opportunity to clarify the modifications introduced in the Grey Wolf Optimization (GWO) algorithm. In response, we have expanded the relevant section in the revised manuscript to provide a detailed explanation of the enhancements made to the standard GWO.  Specifically, our Improved Grey Wolf Optimization (IMGWO) algorithm introduces the following key changes:   1. **Dynamic convergence control:** We incorporated an adaptive parameter adjustment strategy to balance exploration and exploitation during optimization, preventing premature convergence. 2. **Enhanced leader updating mechanism:** The position update equations of alpha, beta, and delta wolves were refined to improve diversity and maintain a more effective search of the solution space. 3. **Hybrid initialization strategy:** Random initialization was replaced with a quasi-oppositional population initialization method to ensure better coverage of the search space and faster convergence.   These modifications collectively enhance the optimization stability and accuracy of parameter tuning for the Extreme Learning Machine (ELM) classifier. The detailed algorithmic description and pseudocode have been added in **Section 3.10.3**, along with comparative performance results between the standard GWO and the proposed IMGWO to demonstrate the improvement.  We appreciate the reviewer’s comment, which helped us clarify and strengthen the methodological explanation. |
| Comment 5 | Please list the exact features in tabular format during each phase , which of the features are selected and which are rejected , any why , if possible |
| Response | Thank you for this thoughtful and detailed suggestion. We acknowledge the importance of discussing. As suggested, the extracted and selected features in a tabular format would enhance the clarity and transparency of our feature selection process.  In response, we have included a comprehensive Table 3 in **Section 4.3** in the revised manuscript. summarizing the features obtained during each phase of the pipeline feature extraction (FDCT-WRP), dimensionality reduction (PCA + LDA), and final selection (IMGWO). The table clearly lists:  The initial extracted features,  The features retained after PCA and LDA based on variance contribution and class separability, and the final selected subset chosen by the IMGWO algorithm for classification.  We have also provided brief justifications for why certain features were retained or discarded primarily based on their discriminative power, redundancy reduction, and contribution to model accuracy.  This addition makes the feature selection process more transparent and supports the interpretability of the proposed framework. We appreciate the reviewer’s valuable suggestion, which has helped improve the manuscript’s technical completeness and clarity. |
| Comment 6 | Addressing issues related to scalability, interoperability, and regulatory compliance would enhance the practical relevance of the conclusions. |
| Response | Thank you for this insightful and constructive suggestion. We fully agree that addressing **scalability, interoperability, and regulatory compliance** is crucial to improving the practical applicability of our proposed framework.  In response, we have expanded the **Conclusion and Future Work Section 5.** to include a detailed analysis of these aspects:   - **Scalability:** We discuss how the proposed CAD system can be adapted for large-scale deployment across diverse clinical settings, emphasizing its modular design and compatibility with distributed computing environments. - **Interoperability:** We highlight the framework’s potential integration with existing hospital information systems (HIS) and picture archiving and communication systems (PACS) through standard medical imaging protocols (e.g., DICOM). - **Regulatory Compliance:** We outline key considerations for aligning with **medical AI regulatory frameworks** such as FDA and CE requirements, including data privacy, model transparency, and validation procedures.   These additions strengthen the manuscript by connecting the research findings to real-world clinical translation and compliance standards. We are grateful for the editor’s guidance, which has helped us enhance the practical relevance and completeness of our study. |
| Comment 7 | Although the paper mentions the need for further validation on larger datasets, it could benefit from a more comprehensive discussion on specific avenues for future research and development. Identifying specific research gaps or unresolved questions would provide a clearer roadmap for advancing the field of this disease, its diagnosis and monitoring using deep/machine learning techniques. |
| Response | Thank you for this thoughtful and constructive comment. We completely agree that a more detailed discussion of future research directions would strengthen the manuscript and provide clearer guidance for advancing glaucoma diagnosis and monitoring using AI-based methods.  In response, we have added a Discussion **Section 4.5.** to include a comprehensive discussion outlining specific avenues for future research, including: Validation on larger and multi-center datasets to improve generalizability across populations and imaging devices. Longitudinal studies to assess disease progression and enable predictive modeling of glaucoma onset.  Integration of multimodal data (e.g., OCT scans, visual field tests, and clinical parameters) to enhance diagnostic accuracy. Development of lightweight, real-time models for deployment in low-resource clinical settings. Exploration of federated and privacy-preserving learning frameworks to ensure secure use of patient data across institutions. Human-in-the-loop systems that incorporate clinician feedback for improved interpretability and trustworthiness. These additions provide a clearer roadmap for advancing research in glaucoma detection and AI-driven ophthalmic diagnostics. We thank the reviewer once again for this valuable suggestion, which has helped us strengthen the depth and forward-looking perspective of the manuscript. |
| Comment 8 | These few recent state-of-the-art studies should be discussed where similar features , ML and feature selection have been employed for GLAUCOMA diagnosis ( A three-stage novel framework for efficient and automatic glaucoma classification from retinal fundus images; An artificial intelligence-based smart system for early glaucoma recognition using OCT images ; Histogram of oriented gradients (HOG)-based artificial neural network (ANN) classifier for glaucoma detection) |
| Response | We sincerely thank for this valuable suggestion and for pointing out recent state-of-the-art studies relevant to our work. We agree that discussing these studies would strengthen the literature review and better contextualize our proposed approach within current research trends.  In response, we have **expanded the Related Work** to include a detailed discussion of the following studies:  [82] Singh, L. K., Khanna, M., Garg, H., Singh, R., & Iqbal, M. (2024). A three-stage novel framework for efficient and automatic glaucoma classification from retinal fundus images. Multimedia Tools and Applications, 83(37), 85421-85481.  [83] Singh, L. K., Garg, H., & Khanna, M. (2021). An artificial intelligence-based smart system for early glaucoma recognition using OCT images. International Journal of E-Health and Medical Communications (IJEHMC), 12(4), 32-59.  [84] Singh, L. K., Garg, H., & Khanna, M. (2022). Histogram of oriented gradients (HOG)-based artificial neural network (ANN) classifier for glaucoma detection. International Journal of Swarm Intelligence Research (IJSIR), 13(1), 1-32.  These additions help position our research more clearly relative to existing methodologies, demonstrating both the novelty and the performance improvements achieved by our approach. We appreciate the reviewer’s insightful recommendation, which has helped us enhance the depth and relevance of our literature review. |
| Comment 9 | How one can ensure that the proposed system is generalizable and will repeat the same performance on other datasets also. |
| Response | We sincerely thank for this important and thoughtful question regarding the generalizability of the proposed system. We fully agree that ensuring consistent performance across different datasets is crucial for real-world clinical applicability.  In response, we have added the **Discussion Section 4.5** by elaborating on several steps taken to enhance and assess the model’s generalizability:   1. **Cross-dataset validation strategy:** Although our main experiments were conducted on the G1020 and ORIGA datasets, we performed **cross-validation and domain variation tests** by partitioning the data to simulate unseen conditions. 2. **Robust preprocessing pipeline:** The proposed image preprocessing and normalization techniques are designed to minimize variations caused by different imaging devices and acquisition settings. 3. **Regularization and optimization:** The inclusion of **PCA, LDA, and IMGWO** ensures dimensionality reduction, feature decorrelation, and parameter stability, reducing overfitting to specific datasets. 4. **Future work direction:** We have explicitly mentioned our plan to validate the model on **external, multi-center, and device-diverse datasets** to further confirm generalizability and robustness.   These clarifications have been added to the revised manuscript to clearly address this concern. We are grateful to the reviewer for highlighting this key aspect, which has helped us improve the rigor and applicability of our study. |

| Comment 1 | 1) Please confirm (a) that you are the photographer of 1, 2, and 4, or (b) provide written permission from the photographer to publish the photo(s) under our CC BY 4.0 license. |
| --- | --- |
| Response | Thank you for your valuable suggestion. We appreciate your observation. We have now added a complete list of photographers of 1, 2, and 4. We have added all images to the revised manuscript.  **Figure 1:**  An overview of the processing stages that form the complete GlaucoXAI pipeline. A stepwise representation of the preprocessing, feature extraction, dimensionality reduction and classification stages that make up the GlaucoXAI model.  Found at DOI: dx.doi.org/10.17504/protocols.io.5qpvod61bg4o/v1[23KB]  **Figure 2:**  A comparison of XAI techniques used to interpret model decisions in glaucoma detection. Each visualization highlights regions and features relevant to distinguishing glaucomatous patterns.  Found at DOI: dx.doi.org/10.17504/protocols.io.36wgqpz13vk5/v1[23KB]  **Figure 3:**  This figure depicts the progression of the convergence factor throughout the optimization routine. The trend shows a gradual decline in fluctuations over successive iterations. It indicates that the model approaches a stable state as training advances. The closing segment of the graph reflects that the optimization reaches a consistent behaviour.  Found at DOI: dx.doi.org/10.17504/protocols.io.4r3l21jzqg1y/v1[23KB]  **Figure 4:**  This figure presents representative retinal images taken from the G1020 and ORIGA datasets. The samples highlight variations in image quality and visual characteristics across both datasets.  Found at DOI: dx.doi.org/10.17504/protocols.io.kqdg31onql25/v1[23KB]  **Figure 5:**  This figure shows how the data is allocated to training and testing sets across the folds. The arrangement confirms that each sample is used once for testing while serving in the training set during the other iterations. The layout demonstrates that every sample is selected once for testing and is included in the training set during the other rounds. It provides a visual understanding of how the rotation of folds ensures full participation of all samples. The arrangement reflects a balanced and systematic evaluation pattern across iterations.  Found at DOI: dx.doi.org/10.17504/protocols.io.bp2l6z6ergqe/v1[23KB]  **Figure 6:**  This figure shows a sample glaucoma fundus image along with the corresponding coefficients produced at scale four of the FDCT process. The visualization highlights how the transform captures important structural and textural patterns related to glaucomatous changes. This figure presents a glaucoma fundus image paired with the coefficients generated at scale four using the FDCT method. The coefficient maps reveal localized texture and directional features extracted from the original image. These patterns provide insight into structural changes commonly associated with glaucoma. The visualization helps illustrate how FDCT captures fine details that support the feature extraction stage of the model.  Found at DOI: dx.doi.org/10.17504/protocols.io.j8nlkyj16g5r/v1[23KB]  **Figure 7:**  The figure illustrates how classification accuracy varies with different feature counts. It demonstrates that accuracy improves as more features are added until it stabilizes at an optimal level. Specifically, this figure details the response of accuracy to varying numbers of selected features during evaluation on the G1020 dataset. Initially, the curve shows a significant improvement with the inclusion of more features, but eventually, it levels off as the model reaches its effective feature range. This indicates the point at which additional features contribute minimally to further gains in accuracy. The trend helps identify the ideal feature count that ensures balanced and efficient performance.  Found at DOI: dx.doi.org/10.17504/protocols.io.rm7vz9de2gx1/v1[23KB]  **Figure 8:**  This figure shows how the classification accuracy varies as different feature counts are tested on the ORIGA dataset. The curve indicates an improvement in accuracy with the inclusion of more features until it reaches a stable This figure shows how the classification accuracy varies as different feature counts are tested on the ORIGA dataset. The curve indicates an improvement in accuracy with the inclusion of more features until it reaches a stable and optimal range. and optimal range.  Found at DOI: dx.doi.org/10.17504/protocols.io.14egnrq1yl5d/v1 [23KB]  **Figure 9:**  This figure illustrates how accuracy evolves over successive epochs during a single training run on the G1020 dataset. The curve shows a steady improvement in performance as the model learns from the data. It reaches a region where accuracy begins to stabilize, indicating convergence of the training process. The pattern provides insight into the number of epochs required for effective learning.  Found at DOI: dx.doi.org/10.17504/protocols.io.8epv5kq55v1b/v1 [23KB]  **Figure 10:**  This figure shows how the loss value decreases over successive epochs during a single training run on the G1020 dataset. The curve demonstrates a consistent reduction in loss as the model learns and adjusts its parameters. It reaches a stable region where further epochs produce only small changes in loss. The pattern indicates the point at which the training process approaches effective convergence.  Found at DOI: dx.doi.org/10.17504/protocols.io.261gekb17g47/v1[23KB]  **Figure 11:**  This figure illustrates how accuracy progresses across successive epochs during a single training run on the ORIGA dataset. The curve shows a gradual increase in accuracy as the model improves its learning. It eventually reaches a stable region where further epochs produce minimal change. The trend helps indicate the number of epochs needed for reliable performance.  Found at DOI: dx.doi.org/10.17504/protocols.io.6qpvrwnyblmk/v1[23KB]  **Figure 12:**  This figure presents the decrease in loss across successive epochs during a single training run on the ORIGA dataset. The curve shows a steady drop in loss values as the model adapts and refines its parameters. It reaches a point where the loss stabilizes, indicating that the training process is nearing convergence. The pattern provides insight into the learning behavior and the number of epochs required for effective optimization.  Found at DOI: dx.doi.org/10.17504/protocols.io.e6nvw4y79lmk/v1[23KB]  **Figure 13:**  This figure shows the confusion matrix generated from the deployed classification scheme evaluated on G1020 dataset. The matrix summarizes correct and incorrect predictions across the classes. It provides a clear view of the model performance in terms of true positives, true negatives, false positives and false negatives. The pattern helps assess the reliability of the scheme for glaucoma classification.  Found at DOI: dx.doi.org/10.17504/protocols.io.n2bvjewmxgk5/v1[23KB]  **Figure 14:**  This figure shows the confusion matrix generated from the deployed classification scheme evaluated on ORIGA dataset. The matrix summarizes correct and incorrect predictions across the classes. It provides a clear view of the model performance in terms of true positives, true negatives, false positives and false negatives. The pattern helps assess the reliability of the scheme for glaucoma classification.  Found at DOI: dx.doi.org/10.17504/protocols.io.5qpvod64bg4o/v1[23KB]  **Figure 15:**  This figure compares the performance of multiple classifiers when evaluated alongside the existing model on the G1020 dataset. The results highlight variations in accuracy and overall effectiveness across the different classification approaches. The comparison helps identify which classifier provides the most reliable outcome for glaucoma detection.  Found at DOI: dx.doi.org/10.17504/protocols.io.3byl464yjgo5/v1 [23KB]  **Figure 16:**  This figure presents a comparison of multiple classifiers evaluated together with the existing model on the ORIGA dataset. The results show differences in accuracy and overall performance among the various classification methods. The comparison helps determine which classifier delivers the most dependable results for glaucoma identification.  Found at DOI: dx.doi.org/10.17504/protocols.io.4r3l21jrqg1y/v1[23KB]  **Figure 17:**  This figure presents a heatmap that shows how accuracy changes when different components of the model are modified or removed. The visual pattern reflects the contribution of each configuration to overall performance. It helps identify the components that have the strongest influence on accuracy across both datasets.  Found at DOI: dx.doi.org/10.17504/protocols.io.kqdg31odql25/v1 [23KB]  **Table 1: Fundus images of Glaucoma Data Sets (Glaucomatous vs. Healthy)**  This table presents representative fundus images from glaucoma and non glaucoma categories across the datasets. The samples show clear visual differences between healthy and glaucomatous eyes. They provide an overview of the image characteristics used for model development and evaluation.  Found at DOI: dx.doi.org/10.17504/protocols.io.yxmvmb44og3p/v1[23KB]  **Table 2: Compilation of hyper parameter specifications for distinct classifiers**  This table lists the key hyper parameter settings used for each classifier in the study. The specifications outline the configuration choices applied during training and evaluation. They provide a clear reference for understanding the parameter differences across the classifiers.  Found at DOI: dx.doi.org/10.17504/protocols.io.x54v95n41l3e/v1[23KB]  **Table 3: Comparative analyses (%) of the deployed model based on PCA with LDA techniques**  This table presents a comparison of performance metrics obtained using the combined PCA and LDA approach. The results highlight the effectiveness of this dimensionality reduction strategy in improving classification outcomes. It provides a clear view of how the deployed model performs relative to other evaluated configurations.  Found at DOI: dx.doi.org/10.17504/protocols.io.j8nlkyjp6g5r/v1[23KB]  **Table 4: The effectiveness of the deployed CAD model using the Retinal G1020 dataset result (%) in 10×5-fold approaches with GWO+ELM**  This table reports the performance outcomes achieved by the CAD model on the G1020 dataset under the 10×5fold evaluation setup. The results reflect the contribution of the GWO plus ELM combination to improving classification accuracy and stability. It provides a clear summary of the model effectiveness across repeated validation cycles.  Found at DOI: dx.doi.org/10.17504/protocols.io.14egnrqoyl5d/v1[23KB]  **Table 5: The effectiveness of the suggested CAD model using the Retinal G1020 dataset result (%) in 10× 5-fold approaches with IMGWO+ELM**  This table presents the performance results achieved by the suggested CAD model on the G1020 dataset using the 10×5 fold validation method. The outcomes show the impact of the IMGWO plus ELM combination in enhancing accuracy and overall classification performance. It provides a detailed summary of the model consistency and reliability across multiple evaluation cycles.  Found at DOI: dx.doi.org/10.17504/protocols.io.5jyl88zo8l2w/v1[23KB]  **Table 6: The effectiveness of the suggested CAD model using the Retinal ORIGA dataset result (%) in 10× 5-fold approaches with GWO+ELM**  This table reports the performance obtained by the suggested CAD model on the ORIGA dataset under the 10×5 fold evaluation scheme. The results show how the integration of GWO plus ELM contributes to improved accuracy and stable classification behavior. It provides a clear summary of the model performance across repeated validation iterations.  Found at DOI: dx.doi.org/10.17504/protocols.io.8epv5kqp5v1b/v1[23KB]  **Table 7: The effectiveness of the suggested CAD model using the Retinal ORIGA dataset result (%) in 10× 5-fold approaches with INGWO+ELM**  This table presents the performance outcomes produced by the suggested CAD model on the ORIGA dataset using the 10×5 fold evaluation method. The results show the influence of the INGWO plus ELM combination on improving accuracy and overall classification quality. This provides a detailed view of the model stability and reliability across multiple validation rounds.  Found at DOI: dx.doi.org/10.17504/protocols.io.261gekb77g47/v1[23KB]  **Table 8: Comparative Analysis (%) employed scheme Glaucoma datasets with different specifications**  This table presents a comparison of the performance results obtained from the employed scheme across multiple glaucoma datasets. The outcomes reflect how varying parameter settings and configurations influence overall classification accuracy. It offers a clear summary of the relative strengths of each specification used in the evaluation.  Found at DOI: dx.doi.org/10.17504/protocols.io.81wgbwe83gpk/v1 [23KB]  **Table 9: Performance analysis of Proposed model with existing CAD models with G1020 and ORIGA datasets**  This table compares the performance of the proposed model with several established CAD approaches across the two retinal datasets. The results show how the proposed method performs relative to existing techniques in terms of accuracy and overall effectiveness. It provides a clear summary of the strengths of the proposed model when evaluated against current CAD solutions.  Found at DOI: dx.doi.org/10.17504/protocols.io.n92ld6xeog5b/v1[23KB] |
| Comment 2 | Figure 1. Please confirm whether you drew the images / clip-art within the figure panels by hand. If you did not draw the images, please provide (a) a link to the source of the images or icons and their license / terms of use; or (b) written permission from the copyright holder to publish the images or icons under our CC BY 4.0 license. Alternatively, you may replace the images with open source alternatives. See these open source resources you may use to replace images / clip-art: - https://commons.wikimedia.org - https://openclipart.org/. |
| Response | Thank you for your message and for bringing this to our attention. Regarding Figure 1, we confirm that all images and graphic elements used in the figure have originally created by us specifically for this study and do not reproduce any copyrighted material. No third-party images, clip-art, or graphics has been used. Therefore, the figure is fully original and compatible with the Creative Commons Attribution 4.0 International (CC BY 4.0) license used by PLOS. We have also updated all images according to PLOS format in revised manuscript.  **Figure 1:**  An overview of the processing stages that form the complete GlaucoXAI pipeline. A stepwise representation of the preprocessing, feature extraction, dimensionality reduction and classification stages that make up the GlaucoXAI model.  Found at DOI: dx.doi.org/10.17504/protocols.io.5qpvod61bg4o/v1[23KB] |
| Comment 3 | The following Figure contains screenshots: Figure 9. We are not permitted to publish these under our CC-BY 4.0 license, websites are usually intellectual property and are copyrighted. This includes peripheral graphics of the web browser such as icons and button. We ask that you please remove or replace it. |
| Response | Thank you for the suggestion. We have updated Figure 9 replaced with Figure 9, Figure 10, Figure 11, Figure 12 with these under our CC-BY 4.0 license, websites are usually intellectual property and are copyrighted in the revised manuscript.  **Figure 9:**  This figure illustrates how accuracy evolves over successive epochs during a single training run on the G1020 dataset. The curve shows a steady improvement in performance as the model learns from the data. It reaches a region where accuracy begins to stabilize, indicating convergence of the training process. The pattern provides insight into the number of epochs required for effective learning.  Found at DOI: dx.doi.org/10.17504/protocols.io.8epv5kq55v1b/v1 [23KB]  **Figure 10:**  This figure shows how the loss value decreases over successive epochs during a single training run on the G1020 dataset. The curve demonstrates a consistent reduction in loss as the model learns and adjusts its parameters. It reaches a stable region where further epochs produce only small changes in loss. The pattern indicates the point at which the training process approaches effective convergence.  Found at DOI: dx.doi.org/10.17504/protocols.io.261gekb17g47/v1[23KB]  **Figure 11:**  This figure illustrates how accuracy progresses across successive epochs during a single training run on the ORIGA dataset. The curve shows a gradual increase in accuracy as the model improves its learning. It eventually reaches a stable region where further epochs produce minimal change. The trend helps indicate the number of epochs needed for reliable performance.  Found at DOI: dx.doi.org/10.17504/protocols.io.6qpvrwnyblmk/v1[23KB]  **Figure 12:**  This figure presents the decrease in loss across successive epochs during a single training run on the ORIGA dataset. The curve shows a steady drop in loss values as the model adapts and refines its parameters. It reaches a point where the loss stabilizes, indicating that the training process is nearing convergence. The pattern provides insight into the learning behavior and the number of epochs required for effective optimization.  Found at DOI: dx.doi.org/10.17504/protocols.io.e6nvw4y79lmk/v1[23KB] |

Regards,

Prof. Sujata Dash

Corresponding Author
