# Supplementary material for: Explainable AI-driven diagnosis model for early glaucoma detection using grey-wolf optimized extreme learning machine approach
Source: PLoS Comput Biol. 2026 May 4;22(5):e1013139. doi: 10.1371/journal.pcbi.1013139 (PMC13160443; doi:10.1371/journal.pcbi.1013139)
Supplement: Attachment — Submitted filename: Response to Reviewers.docx [file pcbi.1013139.s003.docx]

**Compliance/Rebuttal Report**

| **Submission Number:** | (PCOMPBIOL-D-25-00962R1) - [EMID:081836a6a42327a9] |
| --- | --- |
| **Title of the paper:** | Explainable AI-Driven Diagnosis Model for Early Glaucoma Detection Using Grey-Wolf Optimized Extreme Learning Machine Approach |
| **Authors:** | Debendra Muduli, Santosh Kumar Sharma, Sujata Dash, Achyut Shankar, Fahad Alasimd, Mustufa Haider Abidi |

Dear Editor,

We extend our heartfelt thanks for considering our article for resubmission after the review. We have considered all the review’s comments and revised the manuscript. The comments were really useful to improve the technical contents and quality of the paper. All typographical errors have been removed for better readability. The response to each reviewer’s comments is given below for your consideration.

**Journal Requirements**

| **Comment 1** | If the reviewer comments include a recommendation to cite specific previously published works, please review and evaluate these publications to determine whether they are relevant and should be cited. There is no requirement to cite these works unless the editor has indicated otherwise. |
| --- | --- |
| Response | We sincerely thank the editor for the careful evaluation of our manuscript and for providing helpful guidance during the review process. We have carefully considered all editorial comments and reviewer suggestions and have revised the manuscript accordingly to improve clarity, methodological transparency, and overall presentation.  In response to the editor’s recommendation regarding reviewer-suggested citations, we carefully examined the referenced publications and evaluated their relevance to the scope of our study. Relevant works have been incorporated into the revised manuscript where appropriate. References that have not directly aligned with the objectives and methodology of the present work have not included to maintain the focus and coherence of the study.  Overall, the revisions have improved the clarity of the manuscript, strengthened the methodological description, and enhanced the transparency of the evaluation process. We appreciate the valuable feedback provided by the editor and reviewers, which has significantly contributed to improving the quality of the manuscript. |
| **Comment 2** | 1) Your manuscript is missing the following sections: Results, and Methods.  Please ensure all required sections are present and in the correct order. Make sure section heading levels are clearly indicated in the manuscript text, and limit sub-sections to 3 heading levels. An outline of the required sections can be consulted in our submission guidelines here: <https://journals.plos.org/ploscompbiol/s/submission-guidelines#loc-parts-of-a-submission> |
| Response | Thank you for bringing this to our attention. In the revised manuscript, we have carefully reviewed the manuscript structure and ensured that all required sections, including **Methods** and **Results**, are clearly included and presented in the correct order according to the journal’s submission guidelines.  Additionally, section headings and subheadings have been reorganized to follow the recommended structure, and the hierarchy of headings has been clearly indicated while limiting the subsection levels to three as instructed. We have also verified that the manuscript now aligns with the formatting requirements outlined in the journal guidelines. We appreciate the editor’s guidance, which has helped improve the organization and readability of the manuscript. |
| **Comment 3** | In the online submission form, you indicated that your data will be submitted to a repository upon acceptance. We strongly recommend all authors deposit their data before acceptance, as the process can be lengthy and hold up publication timelines. Please note that, though access restrictions are acceptable now, your entire minimal dataset will need to be made freely accessible if your manuscript is accepted for publication. This policy applies to all data except where public deposition would breach compliance with the protocol approved by your research ethics board. If you are unable to adhere to our open data policy, please kindly revise your statement to explain your reasoning and we will seek the editor's input on an exemption. |
| Response | Thank you for highlighting the journal’s open data policy. We acknowledge the importance of data accessibility for research transparency and reproducibility. As indicated in the submission form, Following the reviewer’s recommendation and the journal’s open data policy, we have now deposited the minimal dataset required to reproduce the results in a public repository. The dataset is available at **[https://github.com/Santosh-84ss/IMGWO**] and the link has been included in the revised Data Availability Statement in the manuscript. The minimal dataset required to replicate the findings will be made freely available in accordance with the journal’s open data policy. We appreciate the editor’s guidance and will ensure that the data deposition process is completed without delaying publication. |

**Reviewer - #1**

| Comment 1 | The manuscript now goes beyond purely qualitative visualizations and claims to include quantitative and clinician-centric evaluation of explainability, which is a positive step. However, the current presentation lacks sufficient methodological detail to fully assess the robustness of these claims.  Please clarify the following explicitly in the manuscript: How many clinicians participated in the evaluation? |
| --- | --- |
| Response | Thank you for your constructive feedback regarding the methodological clarity of the clinician-centric evaluation.  We appreciate your observation that additional detail is required to properly assess the robustness of the quantitative explainability analysis. In response, we have revised the manuscript to explicitly state the number of clinicians involved in the evaluation. Specifically, (**two** **ophthalmologists** for qualitative validation) clinicians participated in the assessment process.  This information has now been clearly included in the Methods **Section 3.1.** We have also ensured that the description of the evaluation procedure is presented in greater detail to improve transparency and reproducibility.  We hope that these revisions adequately address your concern. |
| Comment 2 | What was the annotation protocol (e.g., optic disc/cup, RNFL regions, disease-relevant ROIs)? |
| Response | Thank you for your valuable comment requesting clarification of the annotation protocol.  “Reliable ground truth annotation plays a critical role in the development of automated glaucoma screening systems. In retinal fundus analysis, clinically meaningful structures such as the optic disc, optic cup, and regions associated with retinal nerve fiber layer alterations are central to disease assessment. Therefore, establishing a structured annotation protocol guided by ophthalmic expertise is essential to ensure that model learning aligns with clinically relevant features. In this study, annotations were defined according to established diagnostic criteria, with particular attention to optic nerve head characteristics and disease-indicative regions. The annotation framework was designed to maintain consistency, minimize subjectivity, and support both model training and clinician-centric evaluation of explainability outcomes.”  The updated description of the annotation protocol has been added to the **Section I.** to improve transparency and reproducibility.  We hope this clarification adequately addresses your concern. |
| Comment 3 | Which quantitative metrics were used to compare saliency maps with expert annotations (e.g., Dice coefficient, IoU, localization error)? |
| Response | Thank you for this important comment regarding the quantitative evaluation of the saliency maps.  We appreciate the reviewer’s insightful query regarding quantitative evaluation of saliency alignment. In the current study, explicit pixel-level segmentation masks were not available; therefore, overlap-based metrics such as Dice coefficient or IoU were not computed. Instead, explainability assessment was conducted through clinician-centric evaluation, where expert ophthalmologists qualitatively examined whether the highlighted regions corresponded to clinically relevant anatomical structures. We have now clarified this methodological choice in the revised manuscript and explicitly stated that quantitative spatial overlap analysis represents an important direction for future work.  We hope this revision sufficiently addresses your concern and strengthens the methodological transparency of the explainability evaluation. |
| Comment 4 | Were multiple annotators involved, and if so, was inter-rater variability assessed? |
| Response | Thank you for raising this important point regarding annotator involvement and inter-rater variability.  We appreciate the reviewer’s valuable comment. Multiple ophthalmologists participated in the annotation process. Although formal statistical inter-rater agreement metrics were not computed, annotations were independently reviewed, and discrepancies were resolved through joint discussion to reach consensus. We have clarified this procedure in the revised manuscript to ensure methodological transparency. We acknowledge that formal inter-rater variability analysis would further strengthen reliability assessment and consider it an important direction for future work.  These clarifications have been added to improve methodological transparency and reproducibility.  We appreciate your suggestion, which has strengthened the reporting of our annotation protocol. |
| Comment 5 | If the clinician study or quantitative evaluation is limited in scale, this should be clearly stated as a limitation, rather than implied as a comprehensive validation. Strengthening transparency here will significantly improve the credibility of the GlaucoXAI framework. |
| Response | We sincerely thank the reviewer for highlighting this important point. We agree that the scale of the clinician-based evaluation and quantitative analysis should be clearly presented to avoid overinterpretation of the validation results. In the revised manuscript, we have explicitly stated that the clinician study was conducted on a limited sample and should be interpreted as preliminary validation rather than comprehensive clinical verification.  Furthermore, we have revised the relevant sections to carefully moderate claims related to robustness and generalizability. A dedicated limitation paragraph has now been added, acknowledging the restricted evaluation scale and emphasizing that larger multi-center studies with broader clinician participation are necessary for definitive validation of the GlaucoXAI framework.  We believe this clarification strengthens the transparency and credibility of the study. |
| Comment 6 | There are several places in the manuscript, particularly within the explainability discussion, where references to glioma, brain imaging, MRI, or FLAIR images appear. These seem to be residual artifacts from prior work and are not appropriate in the context of glaucoma fundus image analysis. |
| Response | Thank you for carefully identifying this issue. We sincerely apologize for the presence of references to glioma, brain imaging, MRI, and FLAIR images within the manuscript. These statements were inadvertent residual text artifacts from earlier drafting stages and are not relevant to the current study, which focuses exclusively on glaucoma detection using retinal fundus images.  In response to your comment, we have thoroughly reviewed the entire manuscript and removed all inappropriate references to neuroimaging and unrelated medical imaging modalities. The explainability discussion has been carefully revised to ensure that all descriptions, terminology, and interpretations are strictly aligned with glaucoma fundus image analysis and the ophthalmic domain.  We appreciate your attention to detail, which has helped us improve the clarity, consistency, and scientific accuracy of the manuscript. |
| Comment 7 | Carefully review the manuscript to remove or replace all references that are unrelated to retinal fundus imaging and glaucoma. |
| Response | Thank you for this important recommendation. We have carefully reviewed the entire manuscript to identify and correct any content that has unrelated to retinal fundus imaging and glaucoma. As part of this revision, we removed and replaced all inappropriate references to unrelated modalities or diseases (for example, brain imaging, MRI, FLAIR, and glioma), and we ensured that the terminology, clinical context, and explainability discussion are consistently aligned with glaucoma screening using retinal fundus images. These corrections have been applied throughout the manuscript, including the Introduction, Methods, Results, and Discussion sections, to improve clarity and domain consistency. These updated references as follows:  [30] Sharma, S. K., Muduli, D., Rath, A., Dash, S., Panda, G., Shankar, A., & Dobhal, D. C. (2025). Discrete ripplet-II transform feature extraction and metaheuristic-optimized feature selection for enhanced glaucoma detection in fundus images using least square-support vector machine. Multimedia Tools and Applications, 84(23), 26447-26479.  [60] Muduli, D., Kumari, R., Akhunzada, A., Cengiz, K., Sharma, S. K., Kumar, R. R., & Sah, D. K. (2024). Retinal imaging based glaucoma detection using modified pelican optimization based extreme learning machine. Scientific Reports, 14(1), 29660.  [61] Muduli, D., Yaqoob, S. I., Sharma, S. K., Kanade, A. S., Shameem, M., Jangwan, H. S., ... & Zamani, A. T. (2025). Cloud-based optimized deep learning framework for automated glaucoma detection using stationary wavelet transform and improved grey-wolf-optimization with ELM approach. Results in Engineering, 26, 104682.  [63] M. N. Do, M. Vetterli, The finite ridgelet transform for image representation, IEEE Transactions on image Processing 12 (1) (2003) 16–28.  [66] Mahesh, T., & Parthiban, L. (2025, August). An Empirical Study of Quantum Machine Learning for Early Glaucoma Detection Using Retinal Fundus Images. In 2025 12th International Conference on Emerging Trends in Engineering & Technology-Signal and Information Processing (ICETET-SIP) (pp. 1-6). IEEE.  [68] Orhanbulucu, F., Ünlü, M., Sevim, D. G., Gültekin, M., & Latifoğlu, F. (2025). Machine learning-based migraine analysis using retinal vessel diameters from optical coherence tomography: an alternative approach. Neurological Sciences, 46(12), 6651-6659.  [71] Mahesh, T., & Parthiban, L. (2025, August). An Empirical Study of Quantum Machine Learning for Early Glaucoma Detection Using Retinal Fundus Images. In 2025 12th International Conference on Emerging Trends in Engineering & Technology-Signal and Information Processing (ICETET-SIP) (pp. 1-6). IEEE. |
| Comment 8 | Ensure that all XAI discussions, examples, and interpretations are strictly grounded in ophthalmic imaging and glaucoma-specific pathology. |
| Response | Thank you for this valuable and constructive suggestion. In response, we have carefully revised the manuscript to ensure that all discussions, examples, and interpretations related to explainable artificial intelligence are strictly grounded in ophthalmic imaging and glaucoma-specific pathology. The explainability analysis has been reformulated to focus exclusively on clinically relevant retinal structures, particularly the optic disc, optic cup, and cup to disc ratio, as well as their established role in glaucoma assessment. We have removed any domain-general or cross-modality interpretations and ensured that all explanations are framed within the context of retinal fundus imaging. Additionally, the clinical interpretation of saliency maps and quantitative evaluation results has been aligned with established ophthalmic diagnostic criteria.  These revisions have been implemented throughout the explainability sections to enhance clinical relevance, consistency, and scientific rigor. |
| Comment 9 | This correction is important for conceptual coherence and editorial quality. |
| Response | Thank you for emphasizing the importance of this correction. We fully agree that ensuring domain consistency is essential for maintaining conceptual coherence and overall editorial quality. In response, we have carefully revised the manuscript to eliminate inconsistencies and ensure that all content is thematically aligned with glaucoma detection using retinal fundus imaging.  These revisions have improved the clarity, logical flow, and scientific precision of the manuscript. We appreciate your guidance, which has helped strengthen both the coherence and the overall presentation of the work. |
| Comment 10 | The overall readability has improved compared to the original submission, but the manuscript still contains: Redundant explanations (particularly in the methodology and results sections), Overly verbose figure captions, Perform a final language and style pass to improve conciseness and flow. |
| Response | Thank you for your careful evaluation and constructive feedback regarding readability and style. In response to your comment, we have performed a thorough final revision of the manuscript with particular attention to conciseness and clarity. Specifically:   - Redundant explanations in the Methodology and Results sections have been carefully removed or consolidated to avoid repetition while preserving essential technical details. - Overly verbose figure captions have been streamlined to focus on key information necessary for interpretation. - The entire manuscript has undergone a comprehensive language and style review to improve sentence structure, clarity, and logical flow. - These revisions have significantly enhanced readability while maintaining scientific rigor and completeness.   We appreciate your guidance, which has helped us further refine the quality and presentation of the manuscript. |
| Comment 11 | Reduce repetition across sections describing FDCT-WRP, PCA+LDA, and IMGWO-ELM. |
| Response | Thank you for highlighting the issue of repetition across sections describing FDCT-WRP, PCA+LDA, and IMGWO-ELM.  In response, we have carefully revised the manuscript to reduce redundancy in the descriptions of these components. The detailed methodological explanations of FDCT-WRP, PCA+LDA, and IMGWO-ELM are now presented clearly in the Methods section only, while subsequent sections such as Results and Discussion reference these methods concisely without restating their full descriptions.  We have consolidated overlapping explanations, removed repeated technical details, and ensured that each section serves a distinct purpose, namely methodological description, experimental findings, and interpretative analysis.  These revisions have improved clarity, conciseness, and structural coherence throughout the manuscript. |
| Comment 12 | Streamline figure captions so they explain what is shown without repeating large portions of the main text. |
| Response | We appreciate the reviewer’s constructive suggestion. In the revised manuscript, all figure captions have been carefully streamlined to ensure they concisely describe what is depicted without reiterating detailed explanations already provided in the main text. Redundant methodological descriptions have been removed, and captions now focus solely on clarifying the visual content, key elements, and relevant labels necessary for interpretation. We believe this revision improves readability and enhances the overall clarity of the manuscript.  We appreciate your guidance, which has helped us enhance the clarity and editorial quality of the manuscript. |
| Comment 13 | Your justification for using PCA, LDA, and ELM, despite their classical nature, is reasonable and aligns with your goals of efficiency, interpretability, and deployment feasibility. However, this rationale would benefit from a more explicit positioning statement early in the manuscript. |
| Response | We sincerely thank the reviewer for this thoughtful observation. We agree that the rationale for selecting PCA, LDA, and ELM should be positioned more explicitly at an early stage of the manuscript. In the revised version, we have added a clear positioning statement in the Introduction to explain that the choice of these classical methods was intentional and guided by the objectives of computational efficiency, interpretability, and practical deployment feasibility.  We now explicitly emphasize that the contribution of this work does not lie in proposing new standalone learning algorithms, but rather in the strategic integration of efficient feature transformation, lightweight classification, and explainability mechanisms within a unified glaucoma assessment framework. We believe this clarification strengthens the conceptual framing and improves alignment between the methodological choices and the stated research goals. |
| Comment 14 | Clearly emphasize that the novelty lies not in individual components, but in their synergistic integration with IMGWO and a multi-method XAI framework, rather than in proposing new standalone algorithms. |
| Response | Thank you for this important suggestion. We agree that the manuscript should more clearly articulate where the true novelty of the work lies. In response, we have revised the Introduction and Methodology sections to explicitly state that the primary contribution of this study is not the proposal of new standalone algorithms, but the synergistic integration of established components within a unified and optimized framework.  Specifically, we now emphasize that the novelty resides in:  The integration of PCA and LDA for discriminative feature representation,  The optimization of ELM parameters using the Improved Modified Grey Wolf Optimizer (IMGWO), and the incorporation of a multi-method XAI framework for clinically grounded explainability in glaucoma fundus image analysis.  This positioning clarifies that the contribution is methodological integration, optimization strategy, and explainability alignment, rather than the invention of entirely new base algorithms.  We believe this revision strengthens the conceptual framing of the study and improves its clarity regarding scientific contribution. |
| Comment 15 | The added discussion on dataset limitations, domain-shift simulations, and future multi-center validation is appropriate and sufficient for this revision. No additional experiments are required at this stage. However, ensure that claims about robustness and generalization are carefully worded and do not overstate the current empirical evidence. |
| Response | Thank you for your positive assessment of the added discussion on dataset limitations, domain-shift simulations, and future multi-center validation. We appreciate your recognition that the revisions are appropriate for the current stage of the work.  In response to your recommendation, we have carefully reviewed the manuscript to ensure that all statements related to robustness and generalization are precisely and conservatively worded. Any language that could be interpreted as implying comprehensive validation or broad clinical generalizability has been moderated. The claims are now explicitly framed within the scope of the datasets and experimental conditions evaluated in this study. We have added discussion on dataset limitations, domain-shift simulations, and future multi-center validation is appropriate and sufficient for this revision as follows”  “Despite the encouraging performance of the proposed GlaucoXAI framework, several limitations should be acknowledged. The clinician-based evaluation and quantitative analysis were conducted on a relatively limited sample, which may restrict the generalizability of the findings. While the results indicate meaningful alignment between model explanations and clinically relevant features, this validation should be interpreted as preliminary rather than definitive clinical confirmation. Additionally, the study was performed within a controlled experimental setting, and external validation across multi-center datasets with broader demographic variability would further strengthen robustness assessment. Future work will focus on large-scale clinical validation, expanded annotator participation, and cross-institutional evaluation to enhance the reliability and translational applicability of the proposed framework.”  These refinements have been incorporated primarily in the Results and Discussion sections to maintain scientific rigor and avoid overstatement of the empirical evidence.  We thank you for this guidance, which has helped us further strengthen the transparency and credibility of the manuscript. |

**Reviewer - #2**

| Comment 1 | Accepted from my side |
| --- | --- |
| Response | We thank the reviewer for the confirmation and for the constructive feedback provided throughout the review process. We appreciate the reviewer’s positive feedback and the valuable comments provided during the review process, which helped improve the clarity and quality of the manuscript. |

Regards,

Dr. Debendra Muduli.

Associate Professor,

Dept. of Computer Science and Engineering,

C.V. Raman Global University, India-752054
